# Long-term antithrombotic therapy and risk of intracranial haemorrhage from cerebral cavernous malformations: a population-based cohort study, systematic review, and meta-analysis

**DOI:** 10.1016/S1474-4422(19)30231-5

**Published:** 2019-10

**Authors:** Susanna M Zuurbier, Charlotte R Hickman, Christos S Tolias, Leon A Rinkel, Rebecca Leyrer, Kelly D Flemming, David Bervini, Giuseppe Lanzino, Robert J Wityk, Hans-Martin Schneble, Ulrich Sure, Rustam Al-Shahi Salman

**Affiliations:** a Department of Neurology, Amsterdam University Medical Center, Amsterdam, Netherlands; b Edinburgh Medical School, College of Medicine and Veterinary Medicine, University of Edinburgh, Edinburgh, UK; c Centre for Clinical Brain Sciences and Usher Institute of Population Health Sciences and Informatics, University of Edinburgh, Edinburgh, UK; d University of Groningen, University Medical Centre Groningen, Groningen, Netherlands; e Department of Neurosurgery, University of Duisburg-Essen, Essen, Germany; f Department of Neurology, Mayo Clinic, Rochester, MN, USA; g Department of Neurosurgery, Mayo Clinic, Rochester, MN, USA; h Department of Neurosurgery, Inselspital, Bern University Hospital, Bern, Switzerland; i Department of Neurology, Johns Hopkins Hospital, Baltimore, MD, USA; j Department of Neurology, Hôpital Lariboisière, Paris, France

## Abstract

**Background:**

Antithrombotic (anticoagulant or antiplatelet) therapy is withheld from some patients with cerebral cavernous malformations, because of uncertainty around the safety of these drugs in such patients. We aimed to establish whether antithrombotic therapy is associated with an increased risk of intracranial haemorrhage in adults with cerebral cavernous malformations.

**Methods:**

In this population-based, cohort study, we used data from the Scottish Audit of Intracranial Vascular Malformations, which prospectively identified individuals aged 16 years and older living in Scotland who were first diagnosed with a cerebral cavernous malformation during 1999–2003 or 2006–10. We compared the association between use of antithrombotic therapy after first presentation and the occurrence of intracranial haemorrhage or persistent or progressive focal neurological deficit due to the cerebral cavernous malformations during up to 15 years of prospective follow-up with multivariable Cox proportional hazards regression assessed in all individuals identified in the database. We also did a systematic review and meta-analysis, in which we searched Ovid MEDLINE and Embase from database inception to Feb 1, 2019, to identify comparative studies to calculate the intracranial haemorrhage incidence rate ratio according to antithrombotic therapy use. We then generated a pooled estimate using the inverse variance method and a random effects model.

**Findings:**

We assessed 300 of 306 individuals with a cerebral cavernous malformation who were eligible for study. 61 used antithrombotic therapy (ten [16%] of 61 used anticoagulation) for a mean duration of 7·4 years (SD 5·4) during follow-up. Antithrombotic therapy use was associated with a lower risk of subsequent intracranial haemorrhage or focal neurological deficit (one [2%] of 61 *vs* 29 [12%] of 239, adjusted hazard ratio [HR] 0·12, 95% CI 0·02–0·88; p=0·037). In a meta-analysis of six cohort studies including 1342 patients, antithrombotic therapy use was associated with a lower risk of intracranial haemorrhage (eight [3%] of 253 *vs* 152 [14%] of 1089; incidence rate ratio 0·25, 95% CI 0·13–0·51; p<0·0001; *I*^2^=0%).

**Interpretation:**

Antithrombotic therapy use is associated with a lower risk of intracranial haemorrhage or focal neurological deficit from cerebral cavernous malformations than avoidance of antithrombotic therapy. These findings provide reassurance about safety for clinical practice and require further investigation in a randomised controlled trial.

**Funding:**

UK Medical Research Council, Chief Scientist Office of the Scottish Government, The Stroke Association, Cavernoma Alliance UK, and the Remmert Adriaan Laan Foundation.

## Introduction

Cerebral cavernous malformations (CCMs) are the second commonest incidental vascular finding on brain MRI.^1^ CCMs can cause stroke due to either intracranial haemorrhage or non-haemorrhagic focal neurological deficit attributable to the anatomical location of the CCM.^2^ The risk of these strokes is higher for people with CCMs that have already caused an intracranial haemorrhage and for those with a brainstem CCM.^3^

Around a quarter of patients with CCMs could have an indication for antithrombotic (anticoagulant or antiplatelet) therapy for the prevention of occlusive vascular disease.^4^, ^5^, ^6^, ^7^ Anticoagulant therapy can be used for prevention of systemic embolism in patients with atrial fibrillation or venous thromboembolism, and antiplatelet therapy for secondary prevention after ischaemic cerebrovascular and cardiovascular diseases. However, few data are available on the effect of antithrombotic therapy on the risk of intracranial haemorrhage in adults with a CCM, leaving it difficult to create guidelines with strong recommendations,^8^ despite expert opinion that anticoagulation is contraindicated in patients with CCMs on the basis of a case report.^9^

**Research in context**

**Evidence before this study**

Expert opinion recommends against the use of anticoagulant therapy for patients with cerebral cavernous malformations (CCMs) on the basis of a single case report. 2017 guidelines found few data for the risk of antithrombotic (anticoagulant or antiplatelet) therapy use in people with a CCM (class III, level C). We searched Ovid MEDLINE and Embase for systematic reviews, meta-analyses, and articles published before Feb 1, 2019, with no language restrictions, reporting original data for intracranial haemorrhage and person-years of follow-up according to antithrombotic therapy use in patients with a CCM. We used a comprehensive search strategy, limited to humans, combining terms for CCMs and antithrombotic therapy (appendix p 2–3). We also searched international registries and reference lists of relevant publications. We did not find any systematic reviews or meta-analyses, but we found four non-randomised, hospital-based, predominantly retrospective comparative cohort studies. Each study found a non-significant association between antithrombotic therapy use and lower risk of subsequent intracranial haemorrhage.

**Added value of this study**

To our knowledge, this is the first prospective, population-based study investigating this question, in which we found an association between antithrombotic therapy use and a lower risk of intracranial haemorrhage or focal neurological deficit due to a CCM (adjusted hazard ratio 0·12, 95% CI 0·02–0·88; p=0·037). Furthermore, our systematic review and meta-analysis of data for 1342 patients confirmed an association between antithrombotic therapy use and a lower risk of intracranial haemorrhage (incidence rate ratio 0·25, 95% CI 0·13–0·51; p<0·0001).

**Implications of all the available evidence**

We did not find evidence of a harmful association between the use of antithrombotic therapy and intracranial haemorrhage in adults with CCM, which is reassuring when antithrombotic therapy is required by patients with CCMs for other indications. The possibility that antithrombotic therapy might be beneficial for the prevention of intracranial haemorrhage from a CCM requires investigation in a randomised controlled trial.

Some observational studies have found non-significant associations between the use of long-term antithrombotic therapy and a lower risk of intracranial haemorrhage from a CCM,^4^, ^5^, ^6^, ^7^ but these studies were mostly small, retrospective, hospital-based studies that investigated associations with intracranial haemorrhage alone (omitting non-haemorrhagic focal neurological deficits). Both haemorrhagic and non-haemorrhagic focal neurological deficits might be triggered by thrombosis in the CCM or an associated venous malformation,^2^ raising the hypothesis that antithrombotic drugs might benefit patients with CCMs.

We set out to investigate the association between antithrombotic therapy and intracranial haemorrhage or focal neurological deficit in adults with CCMs in a large prospective, population-based cohort study with long-term follow-up. We also sought to maximise the precision of the estimated association between antithrombotic therapy and intracranial haemorrhage from CCMs by doing a systematic review and combining the population-based data with the hospital-based data in a meta-analysis.

## Methods

### Study design and participants in the population-based cohort study

In the population-based cohort study, we used anonymous data from the Scottish Audit of Intracranial Vascular Malformations (SAIVMs), which is an ongoing National Health Service clinical audit of the care and outcome of people aged 16 years or older who were first diagnosed with any type of intracranial vascular malformation during 1999–2003 or 2006–10 while living in Scotland, UK (case ascertainment did not occur 2004–05, but follow-up continued uninterrupted).^10^, ^11^ In the current analysis, we included every individual aged 16 years or older with a first-in-a-lifetime definite diagnosis of CCM identified by the SAIVMs. Details of how the SAIVMs collected the data, recruited, and followed-up individuals in the database have been published previously.^11^ The Multicentre Research Ethics Committee for Scotland (MREC/98/0/48) and the Fife and Forth Valley Research Ethics Committee (08/S0501/76) approved the observational studies (to which an opt-out consent policy applied) and postal questionnaire studies (which required opt-in consent). The study protocol is available online.

### Procedures of the population-based cohort study

We categorised the type of first clinical presentation by the symptoms and signs that led to the initial CCM diagnosis (regardless of earlier events that might, in retrospect, have been attributable to a CCM).^11^ One of three neuroradiologists verified CCM diagnoses with reference to accepted criteria,^12^, ^13^ but if they were not certain about the CCM diagnosis, the MRI was reviewed by another neuroradiologist independently, and a consensus opinion reached on diagnostic certainty. The neuroradiologist collected data for CCM location and imaging evidence of acute, subacute, or chronic intracranial haemorrhage. The inception point was the time of first clinical presentation.^11^ We collected demographic information and medical history from medical records at baseline, and identified treatment and outcomes using annual prospective surveillance of hospital records, primary care practitioner records, and postal questionnaires to both the patients and their primary care practitioners. We retrospectively collected data for antithrombotic therapy use from these data sources. Long-term antithrombotic therapy use was defined as the prescription and receipt of anticoagulant or antiplatelet therapy for at least 90 days at any time after inception, but before the first outcome event or the end of follow-up (if an outcome event did not occur). The primary outcome was a composite of new stroke due to intracranial haemorrhage (confirmed by acute or subacute haemorrhage on brain imaging consistent with the time of symptom onset) or new persistent or progressive focal neurological deficit definitely attributable to the location of the CCM (but without evidence of a new haemorrhage on brain imaging).^2^, ^11^ We included focal neurological deficits due to a CCM because these events are often of similar severity to intracranial haemorrhage due to a CCM; furthermore, focal neurological deficits might be undetected haemorrhages or possibly thrombosis that might be affected by antithrombotic therapy.^11^ We quantified intracranial haemorrhage alone for our secondary outcome to facilitate comparison with other studies. Two investigators assessed outcome events using available clinical, radiological, and pathological information, masked to antithrombotic therapy use. The cause of death was established using death certificates, autopsy records (if post-mortem examination had been done), clinical records, and any brain imaging that had been done.

### Search strategy and selection criteria for the systematic review

We searched Ovid MEDLINE and Embase from database inception until Feb 1, 2019, to identify comparative studies describing the association between antithrombotic therapy use in patients with CCM and intracranial haemorrhage (appendix p 2–3). We also searched the Cochrane Library, ClinicalTrials.gov, International Standard Randomised Controlled Trials Number Registry, and did a manual search of the bibliographies of relevant publications. We considered publications for inclusion if they reported original data for intracranial haemorrhage and person-years of follow-up according to antithrombotic therapy use. We excluded case reports. There was no language restriction. Two authors (SMZ and CRH) did the literature search and quality assessment independently and completed a data extraction form. Any disagreements in the data were resolved by a third reviewer (RA-SS). We extracted data for the following characteristics: publication characteristics, countries or regions of the study, study design, inclusion criteria, patient characteristics, sample size, antithrombotic therapy use and type, duration and completeness of follow-up, and clinical outcomes. We assessed the quality of the observational studies using the Cochrane risk of bias tool. The primary outcome was the occurrence of intracranial haemorrhage, as defined by the study, after CCM diagnosis during all available follow-up.

### Statistical analysis

We compared baseline characteristics and outcomes between patients using or not using antithrombotic therapy in the population-based cohort study to detect potential differences between the two groups. Continuous variables with a normal distribution are reported as the mean and SD or the median and IQR. Categorical variables are reported as percentages with their corresponding 95% CIs. For statistical comparisons between the two groups, we used the χ^2^ test or, in case of low frequencies, Fisher's exact test. For continuous variables, we used an unpaired *t test* or Mann-Whitney *U* test, as indicated. We quantified completeness of the follow-up data we had accrued as a proportion of the potential follow-up that could have been obtained before the end of the timeframe for these analyses.^14^ We used life tables and Kaplan-Meier survival analysis up to 15 years of follow-up, followed by multivariable Cox regression analysis if proportional hazard assumptions were satisfied,^15^ with prespecified adjustment for type of CCM presentation and location of CCM (dichotomised as brainstem [midbrain, pons, or medulla] or other locations).^3^ We also adjusted for age because of the baseline difference between patients according to antithrombotic therapy use. We censored follow-up at CCM treatment with neurosurgical excision or stereotactic radiosurgery, or at death not due to an outcome event.

For each study in the systematic review, we calculated the incidence rate ratio (IRR) of intracranial haemorrhage during the total number of person-years of follow-up for antithrombotic therapy users versus non-users. We generated a pooled estimate by meta-analysis using the inverse variance method and a random effects model. We considered two-sided probability values of less than 0·05 significant. We quantified inconsistency between studies using the *I*^2^ statistic. We analysed all data using Review Manager 5.3 or IBM SPSS Statistics version 25.0.

### Role of the funding source

The funders of this study had no role in the study design, data collection, data analysis, data interpretation, or writing of the report. The corresponding author had full access to all the data in the study and had final responsibility for the decision to submit for publication.

## Results

306 adult residents in Scotland were newly diagnosed with a CCM during 1999–2003 or 2006–10. The median age of the 306 patients at the initial presentation that led to CCM diagnosis was 44·0 years (IQR 32·0–58·0) and 160 (52%) were women. After excluding six adults whose CCM was diagnosed incidentally at autopsy and who did not contribute follow-up data, we included 300 adults in our analyses (table 1). Of these 300 adults, 61 used antithrombotic therapy (ten [16%] of 61 used anticoagulation, alone or in combination with antiplatelet therapy). 32 (53%) of these 61 adults were already using antithrombotic therapy at the time of CCM diagnosis. Patients who used antithrombotic therapy were older, the type of presentation of their CCM was more likely to be incidental and less likely to be with intracranial haemorrhage, and they were more likely to have a history of hypertension, ischaemic heart disease, ischaemic cerebrovascular disease, and atrial fibrillation than patients never using antithrombotic therapy, but there were no significant differences in sex, CCM multiplicity, and CCM brainstem location at baseline (table 1).

**Table 1:** Baseline characteristics of adults in the prospective population-based cohort study, stratified by use of antithrombotic therapy

		**Used antithrombotic therapy after presentation (n=61)**	**Never used antithrombotic therapy after presentation (n=239)**	**p value**
Sex				0·13
	Women	27 (44%)	132 (55%)	..
	Men	34 (56%)	107 (45%)	..
Age, years		57·0 (45·5–65·0)	39·0 (31·0–53·0)	<0·0001
Comorbidities				
	Hypertension	32 (52%)	28 (12%)	<0·0001
	Ischaemic heart disease	24 (39%)	6 (3%)	<0·0001
	Ischaemic stroke or transient ischaemic attack	21 (34%)	4 (2%)	<0·0001
	Atrial fibrillation	7 (11%)	1 (<1%)	<0·0001
	Mechanical heart valve	0	0	..
Type of CCM presentation				
	Incidental	39 (64%)	90 (38%)	<0·0001
	Focal neurological deficit	4 (7%)	27 (11%)	0·28
	Epileptic seizure	13 (21%)	75 (31%)	0·12
	Intracranial haemorrhage	5 (8%)	47 (20%)	0·035
CCM imaging characteristics				
	Multiple CCM	16 (26%)	81 (34%)	0·25
	Brainstem CCM	8 (13%)	26 (11%)	0·62

Data are n (%) or median (IQR). CCMs=cerebral cavernous malformations.

**Figure 1:**
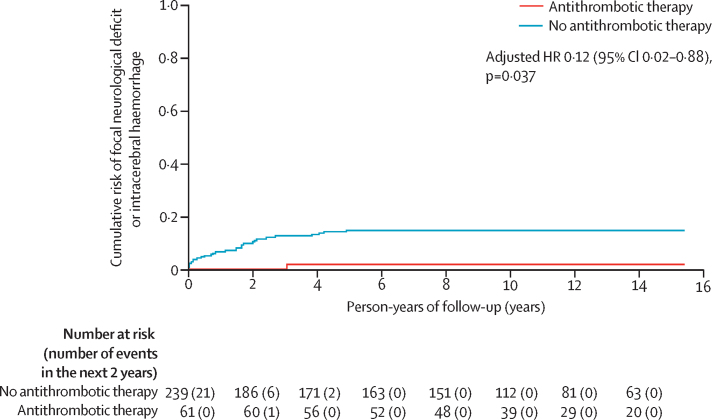
Kaplan-Meier plot Risk of first intracranial haemorrhage or persistent or progressive focal neurological deficit due to cerebral cavernous malformations according to antithrombotic therapy use during 15 years of follow-up in the Scottish Audit of Intracranial Vascular Malformations.

We followed up the 300 adults with a CCM who were alive at initial presentation for the primary outcome of intracranial haemorrhage or focal neurological deficit definitely related to CCM until Mar 7, 2019, for a mean duration of 11·6 years (SD 5·0) until first outcome or censoring (total 3634 person-years of 3843 potential person-years, for an overall median completeness of 95%, IQR 94–99).^14^ All intracranial haemorrhages were intracerebral. The mean duration of antithrombotic therapy use during follow-up was 7·4 years (SD 5·4). One patient using antiplatelet therapy since CCM diagnosis developed the primary outcome while still taking antiplatelet therapy (one [2%] of 61 antithrombotic therapy users during 706 person-years of follow-up), which was less frequent than in patients not using antithrombotic therapy (29 [12%] of 239 during 2208 person-years of follow-up; log-rank p=0·011; figure 1). After confirming the proportional hazards assumption (appendix p 4) and adjusting for age, type of presentation, and CCM location, use of antithrombotic therapy was associated with a lower risk of the primary outcome (adjusted HR 0·12, 95% CI 0·02–0·88; p=0·037; table 2). Post-hoc sensitivity analyses revealed similar, but non-significant associations between antithrombotic therapy and the primary outcome when antithrombotic therapy was a time-dependent covariate (adjusted HR 0·30, 95% CI 0·04–2·32; p=0·25), when the cohort was restricted to brainstem CCMs (adjusted HR 0·16, 0·02–1·28; p=0·084; appendix p 5), when the cohort was restricted to patients presenting with intracranial haemorrhage (adjusted HR 0·41, 0·05–3·21; p=0·40; appendix p 6), when the cohort was restricted to the 29 patients who started antithrombotic therapy after first presentation (unadjusted HR 0·04, 95% CI 0·00–4·87; p=0·189; appendix p 7), when the cohort was restricted to the 228 patients without a brainstem CCM or intracranial haemorrhage at presentation (unadjusted HR 0·033, 0·00–34·94, p=0·34), and when analysing the potential competing risks of death and CCM treatment as outcomes (adjusted HR 0·59, 0·33–1·03; p=0·061; appendix p 8). We were unable to do sensitivity analyses of the primary outcome according to the type of antithrombotic therapy because no patients using anticoagulant therapy had the primary outcome. In analyses of the secondary outcome of intracranial haemorrhage alone (in which one patient, who had not used antithrombotic therapy before developing a focal neurological deficit [primary outcome], used antithrombotic therapy afterwards and was analysed accordingly for the secondary outcome), there was no significant association between antithrombotic therapy use and a lower risk of intracranial haemorrhage (one [2%] of 62 patients during 726 person-years of follow-up *vs* 18 [8%] of 238 during 2342 person-years of follow-up; log-rank p=0·070; appendix p 8–9).

**Table 2:** Cox proportional hazards regression of associations with intracranial haemorrhage or persistent or progressive focal neurological deficit due to CCM during 15 years of follow-up in the Scottish Audit of Intracranial Vascular Malformations

		**Outcome events in sample**	**Log-rank p value**	**Unadjusted hazard ratio**	Adjusted hazard ratio*
CCM location			<0·0001	8·79 (4·29–17·99); p<0·0001	7·14 (3·14–16·27); p<0·0001
	Brainstem	15/34 (44%)	..	..	..
	Non-brainstem	15/266 (6%)	..	..	..
Type of CCM presentation			<0·0001	6·36 (3·10–13·04); p<0·0001	3·20 (1·47–6·97); p=0·0033
	Intracranial haemorrhage	15/52 (29%)	..	..	..
	Other	15/248 (6%)	..	..	..
Age at presentation (per year increase)		NA	NA	1·00 (0·98–1·02); p=0·94	0·99 (0·97–1·02); p=0·53
Antithrombotic therapy use after presentation			0·011	0·12 (0·02–0·85); p=0·034	0·12 (0·02–0·88); p=0·037
	Yes	1/61 (2%)	..	..	..
	No	29/239 (12%)	..	..	..

Data are n/N (%) or hazard ratio (95% CI); p value. CCM=cerebral cavernous malformations. NA=not applicable.

* Adjusted for age, type of CCM presentation, and CCM location.

**Figure 2:**
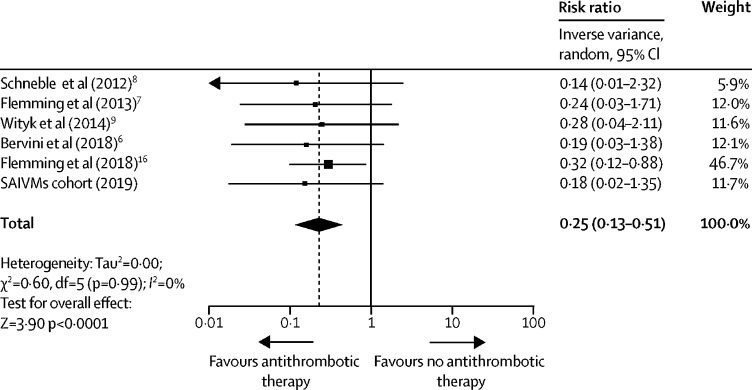
Meta-analysis of the association between antithrombotic therapy use and the risk of intracranial haemorrhage from cerebral cavernous malformations in cohort studies SAIVMS=Scottish Audit of Intracranial Vascular Malformations.

Of the 180 records identified in the systematic review, we selected eight potentially relevant studies, of which four met our inclusion criteria in our meta-analysis (table 2). Studies were exclusively single-centre, hospital-based, non-randomised cohort studies at moderate to high risk of bias (appendix p 10).^4^, ^5^, ^6^, ^7^ We included these four studies, unpublished data from the Mayo Clinic (Rochester, MN, USA),^16^ and the SAIVMs cohort study in a meta-analysis of 1342 patients: 1089 [81%] did not use antithrombotic therapy (152 intracranial haemorrhages occurred during 6214 person-years), 46 [3%] used anticoagulant therapy (one intracranial haemorrhage occurred during 260 person-years), and 207 [15%] used antiplatelet therapy alone (seven intracranial haemorrhages during 1221 person-years; table 3). Baseline imbalances in this larger overall dataset were similar to SAIVMs: patients who used antithrombotic therapy were older, less often women (104 [41%] of 253 *vs* 603 [55%] of 1089; p<0·0001), and presented less often with intracranial haemorrhage (36 [14%] of 253 *vs* 316 [29%] 1089; p<0·0001) than patients who did not use antithrombotic therapy. Antithrombotic therapy use was associated with a lower risk of intracranial haemorrhage (all of which were intracerebral) from a CCM (eight [3%] of 253 *vs* 152 [14%] of 1089, IRR 0·25, 95% CI 0·13–0·51; p<0·0001) with no inconsistency between studies (*I*^2^=0%; figure 2). Of the eight patients using antithrombotic therapy, who had an intracranial haemorrhage during follow-up, one (13%) used anticoagulation and seven (88%) used antiplatelet therapy. In post-hoc sensitivity analyses, we compared patients taking antiplatelet therapy alone with patients not taking antithrombotic therapy (IRR 0·30, 95% CI 0·15–0·62; p=0·0010) and patients taking anticoagulant therapy with patients not taking antithrombotic therapy (IRR 0·53, 0·19–1·52; p=0·24; appendix p 11). Antithrombotic therapy remained associated with a lower risk of intracranial haemorrhage in a post-hoc sensitivity analysis excluding the unpublished Mayo Clinic cohort (IRR 0·16, 0·06–0·42; p=0·00020).

**Table 3:** Characteristics of cohort studies included in the meta-analysis

		**Schneble et al, 2012**^6^**(n=87)**		**Flemming et al, 2013**^5^**(n=292)**		**Wityk et al, 2014**^7^**(n=96)**		**Bervini et al, 2018**^4^**(n=365)**		**Flemming et al, 2018**^16^**(n=202)**		**SAIVMs cohort (n=300)**	
		Never used ATT	Ever used ATT	Never used ATT	Ever used ATT	Never used ATT	Ever used ATT	Never used ATT	Ever used ATT	Never used ATT	Ever used ATT	Never used ATT	Ever used ATT
N		71 (82%)	16 (18%)	252 (86%)	40 (14%)	74 (77%)	22 (23%)	294 (81%)	71 (19%)	160 (79%)	42 (21%)	238 (79%)	62 (21%)
Sex													
	Women	44 (62%)	6 (38%)	135 (54%)	19 (48%)	49 (66%)	9 (41%)	150 (51%)	19 (27%)	94 (59%)	23 (55%)	131 (55%)	28 (45%)
	Men	27 (38%)	10 (62%)	117 (46%)	21 (52%)	25 (34%)	13 (59%)	144 (49%)	52 (73%)	66 (41%)	19 (45%)	107 (45%)	34 (55%)
Mean age, years (SD)		54·0 (16·5)	68·4 (16·1)	43·2 (18·6)	62·4 (13·2)	38·2 (15·9)	53·0 (15·5)	46·8 (18·2)	62·4 (15·8)	41·9 (15·7)	51·4 (16·2)	42·2 (15·1)	54·5 (14·9)
Presentation with haemorrhage		11 (15%)	0	69 (27%)	5 (13%)	23 (31%)	5 (23%)	102 (35%)	14 (20%)	64 (40%)	6 (14%)	47 (20%)	5 (8%)
Multiple CCM		27 (38%)	5 (31%)	51 (20%)	4 (10%)	17 (23%)	8 (36%)	48 (16%)	7 (10%)	45 (28%)	11 (26%)	81 (34%)	16 (26%)
Brainstem CCM location		20 (28%)	3 (19%)	21 (8%)	7 (18%)	21 (28%)	9 (41%)	57 (19%)	8 (11%)	52 (33%)	9 (21%)	25 (11%)	9 (15%)
Haemorrhage during follow-up		9 (13%)	0	31 (12%)	1 (3%)	14 (19%)	1 (5%)	33 (11%)	1 (1%)	47 (29%)	4 (10%)	18 (8%)	1 (2%)
Person-years of follow-up		205	82	1776	247	468	122	813	134	609	170	2342	726

Data are n (%) unless specified. CCM=cerebral cavernous malformations. ATT=antithrombotic therapy. SAIVMs=Scottish Audit of Intracranial Vascular Malformations.

## Discussion

Antithrombotic therapy use was associated with a lower risk of intracranial haemorrhage or focal neurological deficit during long-term follow-up in a prospective population-based study of patients with CCMs, and antithrombotic therapy was associated with a lower risk of intracranial haemorrhage alone in a meta-analysis of six comparative cohort studies of patients with CCMs, with no inconsistency between their findings.

These findings are consistent with previous, non-significant associations observed in individual cohort studies of antiplatelet therapy for patients with CCM.^5^, ^6^, ^7^ The addition of patients using anticoagulant therapy, and the paucity of intracranial haemorrhage outcomes among them, provides further reassurance about the use of any type of antithrombotic therapy for patients with an indication for these drugs, who also have a CCM.

The association between antithrombotic therapy and a lower risk of intracranial haemorrhage or focal neurological deficit from a CCM is consistent with the hypothesis that these events might be triggered by thrombus formation in the dilated caverns of CCMs in which blood flow is slow, or thrombus in an associated venous malformation.^17^, ^18^ A similar pathophysiological mechanism underlies haemorrhagic infarcts in patients with cerebral venous thrombosis,^19^, ^20^ which is treated with anticoagulation to improve outcome and reduce the risk of recurrence, regardless of the presence of haemorrhagic infarction.^21^ Furthermore, antithrombotic therapy had similar, unexpected effects after intracerebral haemorrhage in the RESTART trial (ISRCTN71907627),^22^ which excluded all but a very modest increase in the risk of recurrent intracerebral haemorrhage with antiplatelet therapy for patients taking antithrombotic therapy for the prevention of occlusive vascular disease when they developed intracerebral haemorrhage.

Our study has strengths, including a prospective, population-based cohort design with long-term follow-up and outcome assessment masked to antithrombotic therapy use. Furthermore, we evaluated associations using all known available data, in a comprehensive systematic review and meta-analysis. Moreover, the meta-analysis had a large sample size, many outcomes, and a large number of person-years of follow-up to evaluate the association with precision, which was highly consistent between studies.

The non-randomised designs of all available studies resulted in systematic differences between patients who did, or did not, use antithrombotic therapy during follow-up. These differences were likely to be, at least in part, due to selection bias (eg, patients without a history of intracranial haemorrhage were more likely to use antithrombotic therapy) and confounding by indication (eg, patients who used antithrombotic therapy were older, with a higher likelihood of the competing risk of death). However, we had prespecified statistical adjustment of the findings in our population-based study for two of these potential confounders, and also adjusted for the imbalance in age, after which the associations we found remained significant. Although the findings of the cohort study were not significant in a variety of post-hoc sensitivity analyses, the directions of the associations remained the same (with no suggestion of harm associated with antithrombotic therapy).

In the meta-analysis, we were unable to adjust for baseline imbalances, or explore risk in relation to either familial versus sporadic CCM or the time between presentation with intracranial haemorrhage and starting antithrombotic therapy, because we used aggregate, rather than individual patient data. Some of the studies were retrospective, which might have resulted in recall and information biases. Many of the patients might have used statins as secondary prevention for a cardiovascular or cerebrovascular disease, which have been suggested to reduce the risk of intracranial haemorrhage from CCM in animal models,^23^ although this effect has not been seen in humans.^24^ The small number of patients taking anticoagulant therapy in these studies (probably because of clinicians' fears about these drugs in patients with a CCM) gave imprecise estimates of the association between anticoagulant therapy and intracranial haemorrhage during follow-up.

Our findings have implications for clinical practice. Although a review recommended that anticoagulation was contraindicated for people with CCM,^3^ our findings do not support this recommendation. The associations we have found are reassuring for the use of both types of antithrombotic therapy in clinical practice, in which patients with a CCM have other strong indications for their use to prevent occlusive vascular disease. The possibility that antithrombotic therapy reduces the risk of intracranial haemorrhage and focal neurological deficit from a CCM, perhaps by preventing thrombus formation that might trigger these events, raises a hypothesis that should be tested in a planned randomised controlled trial in people with CCM with or without other indications for antithrombotic therapy.

In summary, we did not find any evidence of a harmful association between the use of antithrombotic therapy and intracranial haemorrhage from a CCM in the population-based study and hospital-based studies, which included a range of patients with sporadic and familial CCM. The possibility that antithrombotic therapy might be beneficial for the prevention of intracranial haemorrhage from CCMs should be investigated in a randomised controlled trial.

## Data sharing

Written proposals will be assessed by a representative of each cohort study and a decision made about the appropriateness of the use of data. A data sharing agreement will be put in place before any data will be shared. Contact the corresponding author for information.
