# Supplementary material for: Long-term antithrombotic therapy and risk of intracranial haemorrhage from cerebral cavernous malformations: a population-based cohort study, systematic review, and meta-analysis
Source: Lancet Neurol. 2019 Oct;18(10):935–41. doi: 10.1016/S1474-4422(19)30231-5 (PMC6744367; doi:10.1016/S1474-4422(19)30231-5)

THE LANCET
Neurology

Supplementary appendix

This appendix formed part of the original submission and has been peer reviewed. We post it as supplied by the authors.

Supplement to: Zuurbier SM, Hickman CR, Tolias CS, et al. Long-term antithrombotic therapy and risk of intracranial haemorrhage from cerebral cavernous malformations: a population-based cohort study, systematic review, and meta-analysis. *Lancet Neurol* 2019; published online Aug 7. http://dx.doi.org/10.1016/S1474-4422(19)30231-5.

ONLINE APPENDIX

Susanna M. Zuurbier, Charlotte R. Hickman, Christos S. Tolias, Leon A. Rinkel, Rebecca Leyrer, Kelly D. Flemming, David Bervini, Giuseppe Lanzino, Robert J. Wityk, Hans-Martin Schneble, Ulrich Sure, and Rustam Al-Shahi Salman for the Scottish Audit of Intracranial Vascular Malformations Steering Committee and Collaborators. Association between long-term antithrombotic therapy and risk of haemorrhage from cerebral cavernous malformations: a population-based cohort study, systematic review, and meta-analysis.

Literature search strategies

OVID Medline

1. Hemangioma, Cavernous, Central Nervous System/
2. Hemangioma, Cavernous/
3. (cavernous (angioma or hemangioma or malformation))
4. cavernoma
5. 2 or 3 or 4
6. brain/ or central nervous system/ or exp cerebral arteries/
7. brain neoplasms/
8. (brain or cerebral or intracerebral or central nervous system or intracranial or cerebellar or intraventricular or supratentorial).tw.
9. 6 or 7 or 8
10. 5 and 9
11. 1 or 10
12. Anticoagulant.tw.
13. antithrombotic.tw.
14. antiplatelet.tw.
15. 12 or 13 or 14
16. 11 and 15

OVID EMBASE

1. Brain Hemangioma/
2. brain ventricle cavernoma/
3. cavernous hemangioma/
4. cavernous.tw.

5. cavernoma.tw.

6. 3 or 4 or 5

7. central nervous system/ or exp brain/ or exp brain ventricle/ or exp brain artery/

8. brain tumor/

9. (brain or cerebral or intracerebral or central nervous system or intracranial or cerebellar or intraventricular or supratentorial).tw.

10. 7 or 8 or 9

11. 6 and 10

12. 1 or 2 or 11

13. Anticoagulant agent/

14. antiplatelet.mp

15. 13 or 14

16. 12 and 15

Cox Regression log minus log function to check proportional hazards assumption for the analysis of the primary outcome in the SAIVMs cohort study

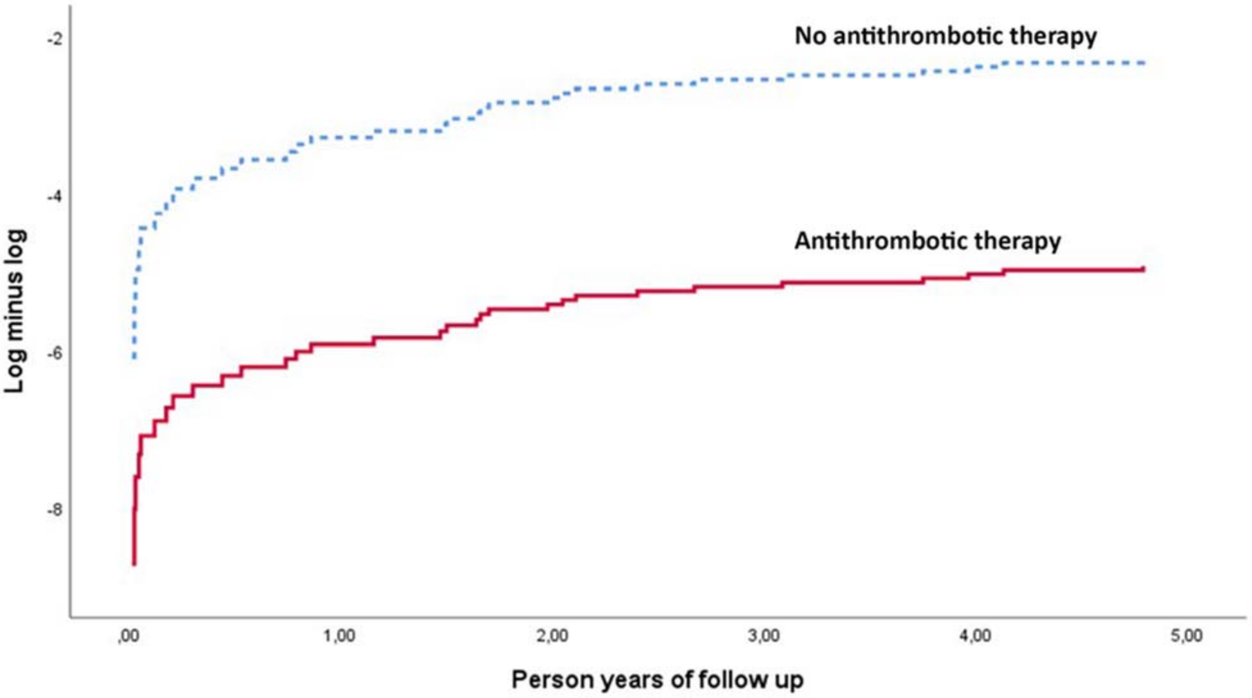

Kaplan Meier plot of the risk of first intracranial haemorrhage or persistent/progressive focal neurological deficit due to CCM according to antithrombotic therapy use, restricted to patients with brainstem CCM, during 15 years of follow-up in the Scottish Audit of Intracranial Vascular Malformations

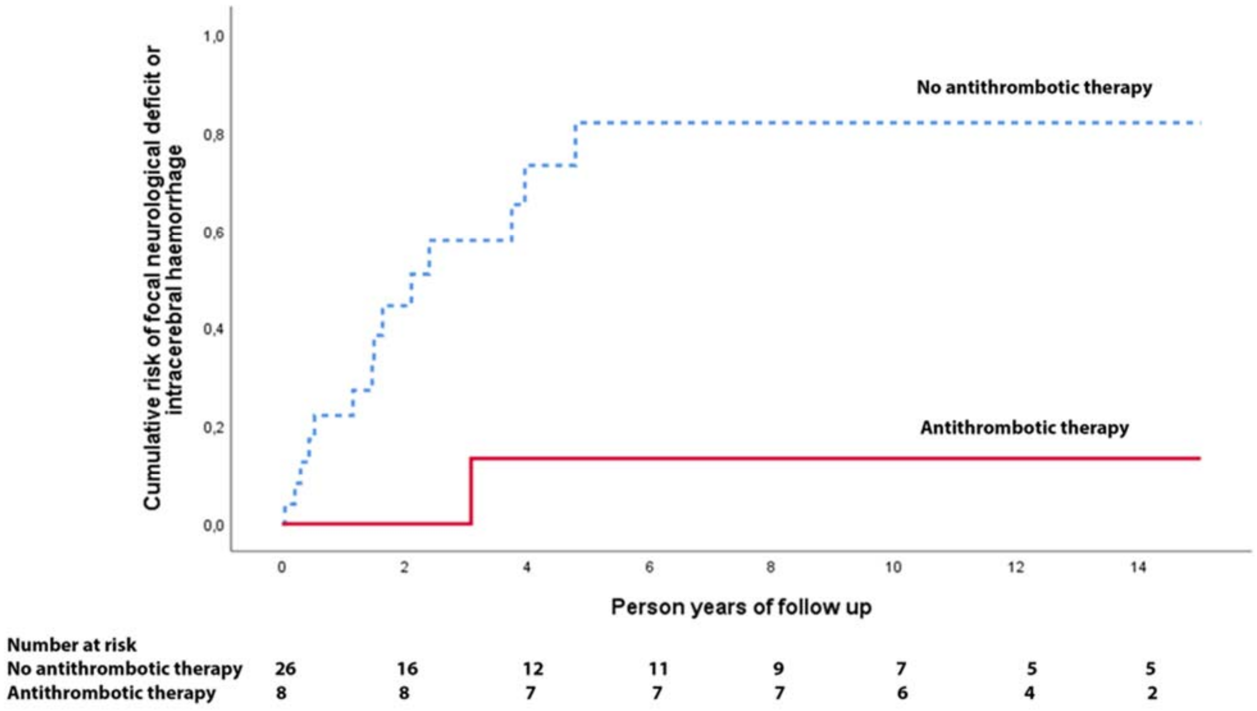

Kaplan Meier plot of the risk of first intracranial haemorrhage or persistent/progressive focal neurological deficit due to CCM according to antithrombotic therapy use, restricted to patients presenting with intracranial haemorrhage, during 15 years of follow-up in the Scottish Audit of Intracranial Vascular Malformations

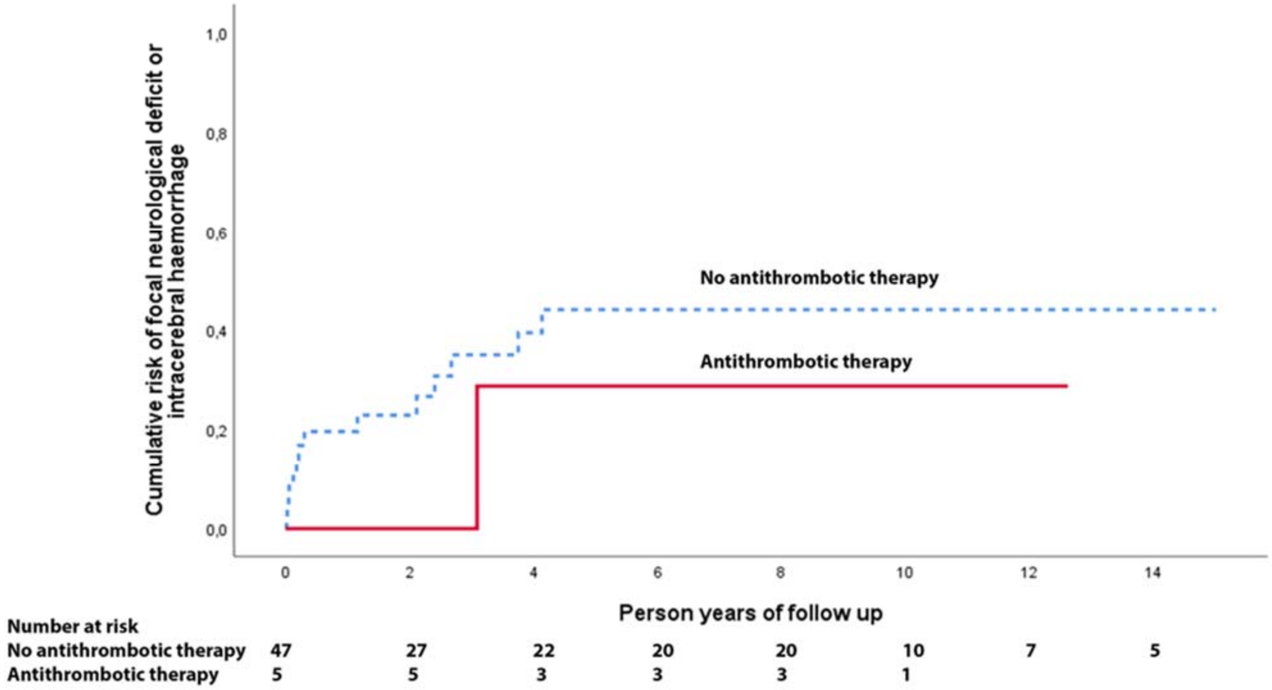

Kaplan Meier plot of the risk of first intracranial haemorrhage or persistent/progressive focal neurological deficit due to CCM according to antithrombotic therapy use, restricted to 29/61 patients starting antithrombotic therapy after first presentation, during 15 years of follow-up in the Scottish Audit of Intracranial Vascular Malformations

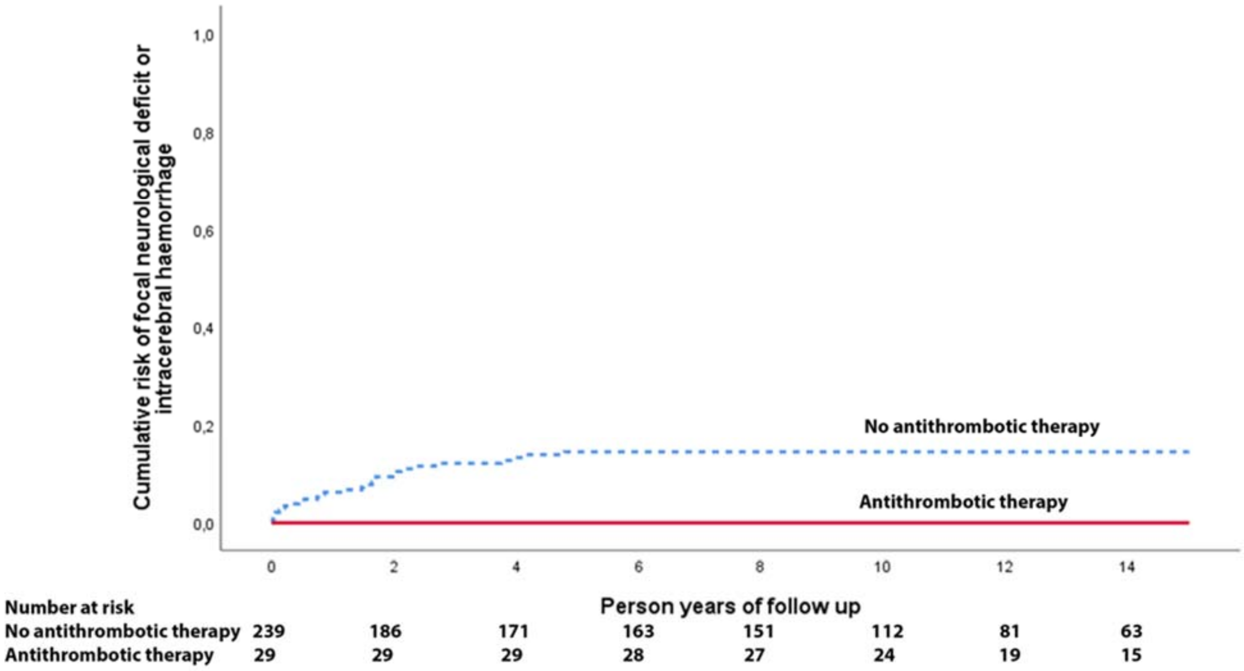

Kaplan Meier plot of the risk of first intracranial haemorrhage or persistent/progressive focal neurological deficit due to CCM, as well as death or first CCM treatment (to explore these as competing risks), during 15 years of follow-up in the Scottish Audit of Intracranial Vascular Malformations

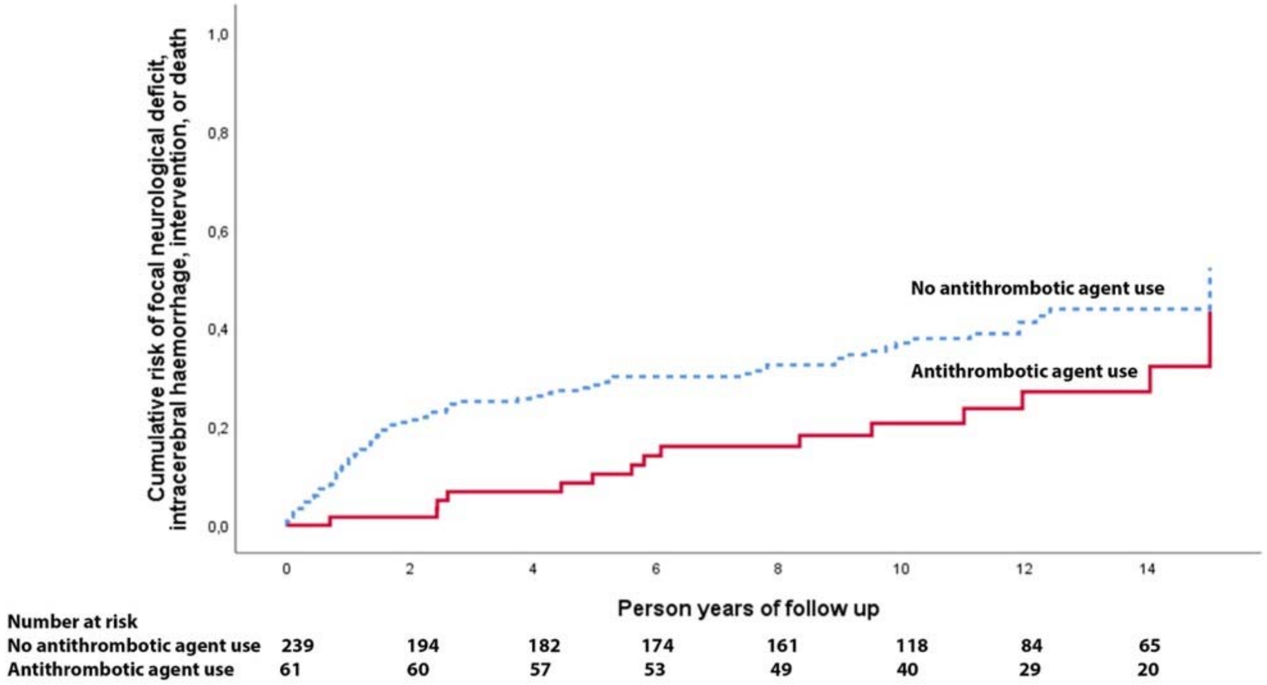

Kaplan Meier plot of the risk of first intracranial haemorrhage due to CCM according to antithrombotic therapy use during 15 years of follow-up in the Scottish Audit of Intracranial Vascular Malformations

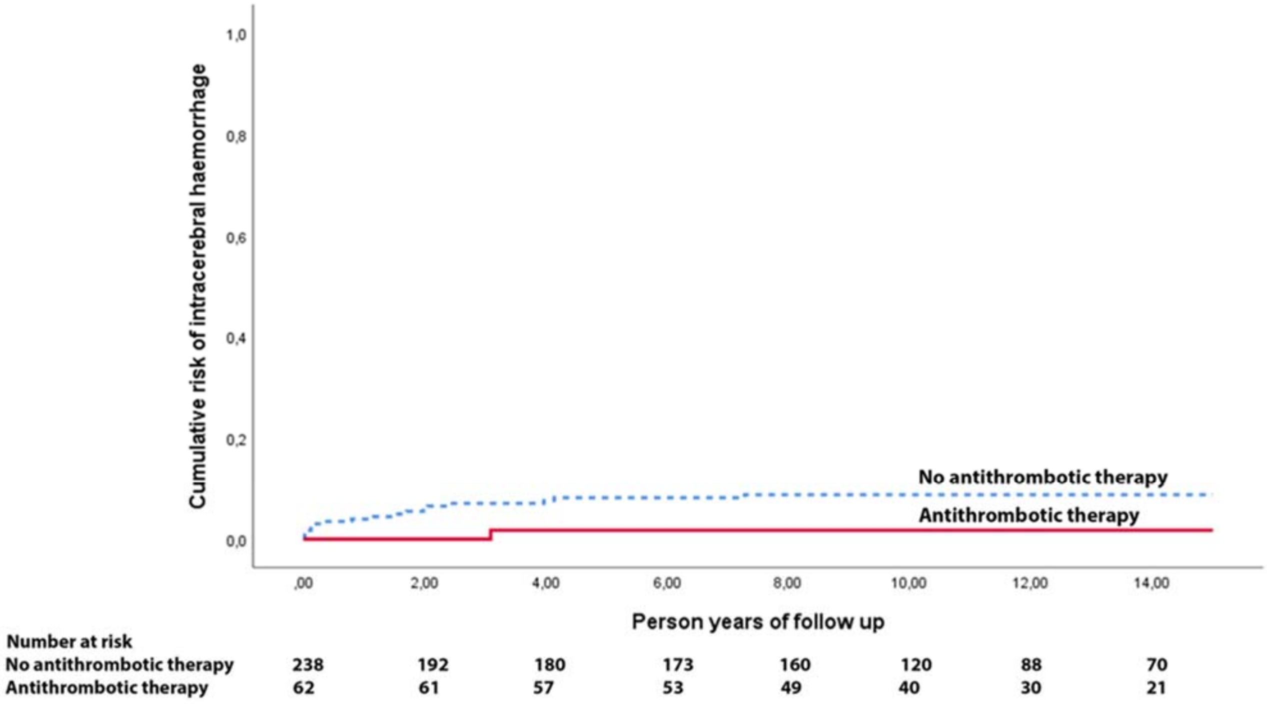

Characteristics of studies included in the meta-analysis

Study characteristics	Bervini	Flemming	Schneble	Wityk	Lanzino	Al-Shahi Salman
**Study design**	Hospital based	Hospital based	Hospital based	Hospital based	Hospital based	Population based
**Period of inclusion**	1980-2015	1989-1999	2008-2010	1987-2009	2015-2019	1999-2003 or 2006-2010
**Identification of patients**	Prospective	Retrospective	Prospective	Retrospective	Prospective	Prospective
**Follow-up of patients**	Retrospective	Retrospective	Prospective and retrospective	Retrospective	Prospective	Prospective
**Random sequence generation**	No	No	No	No	No	No
**Allocation concealment**	No	No	No	No	No	No
**Participants and personnel masked to antithrombotic therapy use**	No	No	No	No	No	No
**Outcome assessment masked to antithrombotic therapy use**	No	No	No	No	Yes	Yes

Post hoc meta-analyses of the association between antiplatelet therapy or anticoagulant therapy use and the risk of intracranial haemorrhage from CCM in cohort studies

Post hoc meta-analysis of antiplatelet therapy alone (n=207) versus no antithrombotic therapy (n=1,089):

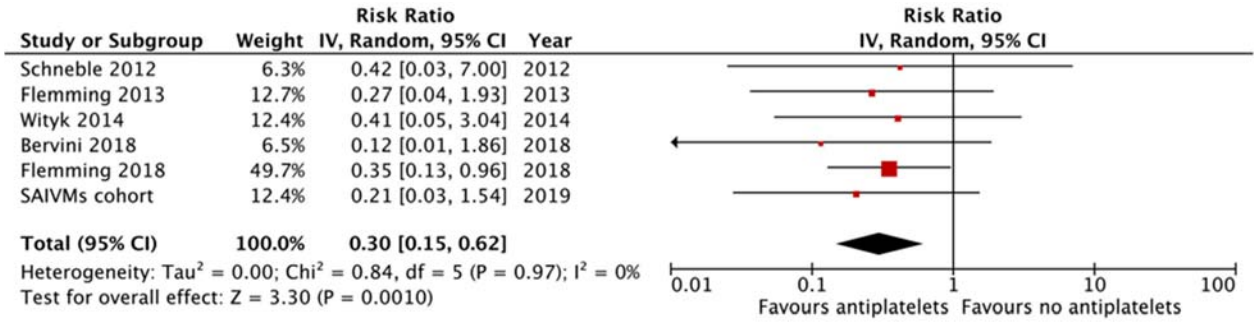

Post hoc meta-analysis of anticoagulant therapy (n=46) versus no antithrombotic therapy (n=1,089):

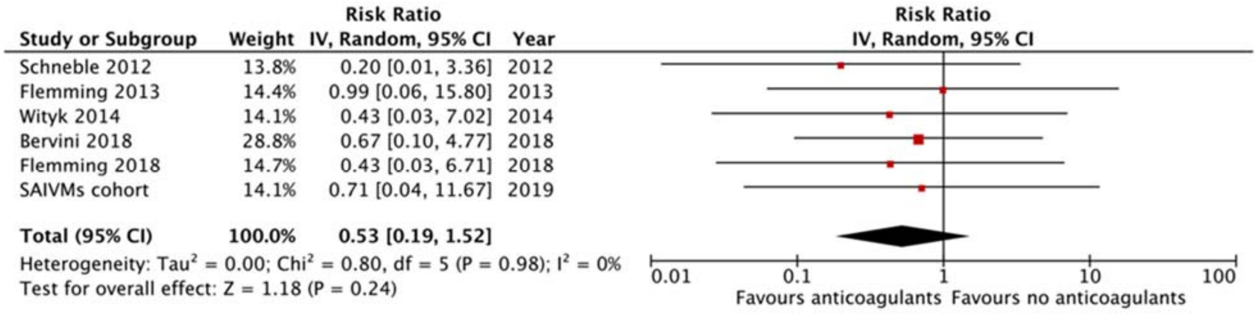

Supplement: Supplementary appendix [file mmc1.pdf]
